# VirtualMicroscopy: ultra-fast interactive microscopy of gigapixel/terapixel images over internet

**DOI:** 10.1038/srep14069

**Published:** 2015-09-11

**Authors:** Ching-Wei Wang, Cheng-Ta Huang, Chu-Mei Hung

**Affiliations:** 1 Graduate Institute of Biomedical Engineering, National Taiwan University of Science & Technology, Taiwan; 2 Department of Biomedical Engineering, National Defence Medical Center, Taiwan

## Abstract

As digital imaging technology advances, gigapixel or terapixel super resolution microscopic images become available. We have built a real time virtual microscopy technique for interactive analysis of super high resolution microscopic images over internet on standard desktops, laptops or mobile devices. The presented virtual microscopy technique is demonstrated to perform as fast as using a microscopy locally without any delay to assess gigapixel ultra high resolution image data through wired or wireless internet by a Tablet or a standard PC. More importantly, the presented technology enables analysis of super high resolution microscopic image across sites and time and allows multi-person analysis at the same time, which greatly speed up data analysis process and reduces miscommunication among scientists and doctors. A web site has been created for illustration purposes. (http://www-o.ntust.edu.tw/~cweiwang/VirtualMicroscopy).

Super high-resolution digital microscopic images are necessary for accurate diagnosis and analysis in many applications such as neurobiology and histopathology, and it is expected to play a revolutionary role to describe high-resolution anatomy of large biological specimens. Recent computer vision studies have been working on capturing and reconstructing large-scale images. Saalfeld *et al.*^1^ introduced an automatic registration method for stitching tiled serial section microscopic stacks together. There have been some works on interactive display of large-scale image. Kopf *et al.*^2^ introduced a method for capturing and interactively displayed gigapixel images, which was released as Microsoft HDview^3^. Leica Aperio WebScope^4^,^5^ has been released as a web based and platform independent digital slide viewer. However, the limitation on the bandwidth to a server over wired or wireless internet, including 3G or 4G mobile telecommunication technologies, makes the retrieval of an extremely large scale image, even compressed, very time consuming^6^, and existing techniques such as Microsoft HDview and Leica Aperio WebScope suffer from significant time delay and image blurriness (see Fig. 1, supplementary video 1 and supplementary video 2). In addition, none of the aforementioned approaches enables real time interaction over internet as using a microscope.

High-resolution image database is extremely large in size, making them expensive to transmit and process, and difficult to scan and search for specific contents. Due to the enormous size of microscopic sections, it is difficult to handle the images efficiently^7^. In evaluation of two state of the art systems^3^,^4^, all suffer from unsatisfactory time delay. Although whole-slide digital imaging has been receiving increased attention in anatomic pathology, due to technology limitation, it is still not widely used in a diagnostic setting^8^. Challenges in high-resolution whole slide images include not only making the image quality good enough but also the interactive visualization speed fast enough to be comparable to the experience of using a microscope^9^.

In this work, we have built a real time virtual microscopy technique for interactive analysis of super high resolution microscopic images over internet. The presented virtual microscopy technique is demonstrated to perform as fast as using a microscopy locally without any delay. More importantly, the presented technology enables analysis of super high resolution microscopic image across regions and time and allows multi-person analysis at the same time, which greatly speed up data analysis process and reduces miscommunication among scientists and doctors. In comparison with the state of the art methods, including Microsoft HDview^3^ and Leica Aperio WebScope^4^, the presented technique is significantly faster (*p* < 0.001 according to Tukey’s HSD and LSD tests). A virtual microscopy demonstration on a standard PC through wired internet is given in the supplementary video 3, and a demonstration on a Tablet through wireless internet is given in the supplementary video 4.

## Results

Two state of the art systems, including Microsoft HDview^3^ and Leica Aperio WebScope^4^, were adopted as benchmark techniques for comparison. As it is impossible to obtain the same images to test in each benchmark system, images in the benchmark systems with the closest dimension were chosen to test, and the size of the super resolution images in the individual benchmark systems is 3.7 GB (the largest image in Microsoft HDview Demo Website) and 12.78 GB (Leica Aperio WebScope), respectively. In evaluation of the proposed method, a full phase lung tissue section was digitalized at 40× magnification using Leica Aperio Scanscope CS2, and the size of the resulting super resolution microscopic image is 12.8 GB with image dimension 84570 × 54248.

In evaluation, four quantitative measurements were set up by adopting two kinds of data assessing tests and two different client site settings. Regarding the data assessing tests, one data assessing test is in Z direction by zooming in the image four times, and the other data assessing test is in X direction by dragging the image from right to left four times. The two client site settings include a standard PC through wired internet (CPU: Intel Core i7-2600 3.4 GHz, RAM: 12 GB, OS: Windows 7 64-bit) and a Tablet through wireless 3G telecommunication (Samsung Note 10.1, CPU: Exynos4412 1.4 GHz, RAM: 2 GB, OS: Android 4.1.1). For the server site of the presented system, instead of using a super computer, a standard PC (CPU: Intel Core i7-2600 3.4 GHz, RAM: 12 GB) is used as the hardware platform to test the performance of the presented method. Statistical analysis was performed using SPSS software^10^, and the quantitative results were analyzed with the Tukey’s Honestly Significant Difference test (Tukey’s HSD) and the Fisher’s Least Square Difference test (LSD) with the significance level 0.001.

### Z-direction test using PC with wired internet

**Figure 2:**
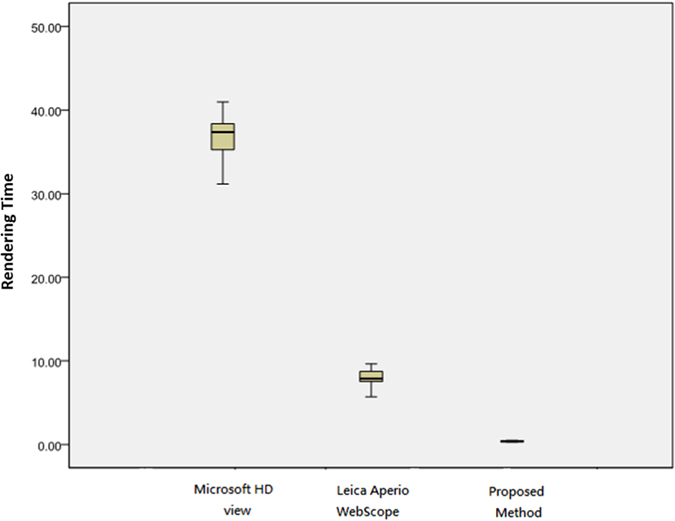
Rendering time by Z-direction test using PC with wired internet. The rendering time of the proposed method performs faster than Microsoft HDview about 94.125 times and Leica Aperio WebScope about 20.352 times.

Table 1 presents the rendering time by Z-direction test using a PC with wired internet, and a statistical analysis using the Tukey’s HSD and the LSD tests is given in Table 2. The experimental results show that the proposed real time method achieves 0.392 seconds averaged rendering time and significantly outperforms the existing methods on Tukey’s HSD and LSD tests (*P* ≤ 0.001). On average, it costs Microsoft HDview and Leica Aperio WebScope 36.897 and 7.978 seconds, respectively, showing that the existing techniques perform poorly and suffer from serious time delay. Overall, the proposed method performs 94.13 times faster than Microsoft HDview and 20.35 times faster than Leica Aperio WebScope on average. A box plot of the quantitative evaluation result is provided in Fig. 2, showing that the proposed method works constantly well than two existing methods overall.

### X-direction test using PC with wired internet

**Figure 3:**
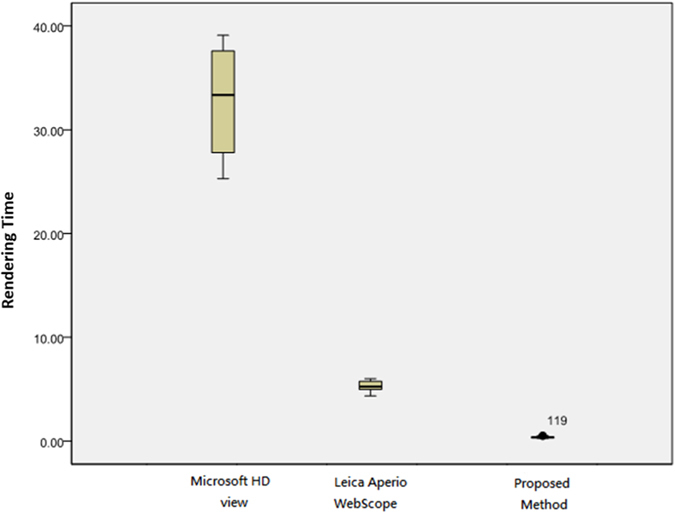
Rendering time by X-direction test using PC with wired internet. The rendering time of the proposed method performs faster than Microsoft HDview about 90.008 times and Leica Aperio WebScope about 14.674 times.

Table 3 presents the rendering time by X-direction test using PC with wired internet. A statistical analysis using the Tukey’s HSD and the LSD tests is given in Table 4. The experimental results show that the proposed real time method achieves 0.363 seconds averaged rendering time and significantly outperforms the existing methods on Tukey’s HSD and LSD tests (*P* ≤ 0.001). On average, it costs Microsoft HDview and Leica Aperio WebScope 32.673 and 5.317 seconds, respectively, showing that the existing techniques perform poorly and suffer from serious time delay. Overall, the proposed method performs 90.01 times faster than Microsoft HDview and 14.67 times faster than Leica Aperio WebScope on average. A box plot of the quantitative evaluation result is provided in Fig. 3, showing that the proposed method works constantly well than the benchmark methods overall.

For the third and fourth quantitative evaluation, a Tablet with wireless internet is adopted as the hardware platform. As Microsoft HDview does not support the Android system, Microsoft HDview can not function on the Tablet (Samsung Note 10.1), and therefore comparison is performed between the presented system and the Leica Aperio WebScope.

### Z-direction test using a Tablet with wireless internet

**Figure 4:**
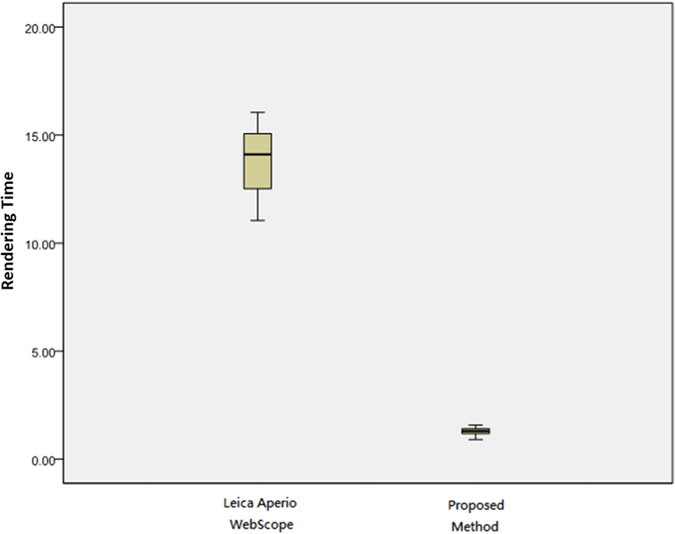
Rendering time by Z-direction test using a Tablet with wireless internet. The rendering time of the proposed method performs faster than Leica Aperio WebScope about 9.933 times.

Table 5 presents the rendering time by Z-direction test using a Tablet with wireless internet, and a statistical analysis using the Tukey’s HSD and the LSD tests is given in Table 6. The experimental results show that the proposed method achieves 1.39 seconds averaged rendering time and significantly outperforms the benchmark method on Tukey’s HSD and LSD tests (*P* ≤ 0.001). On average, it costs Leica Aperio WebScope 13.85 seconds, showing that the existing technique performs poorly and suffers from serious time delay. Overall, the proposed method performs 9.93 times faster than Leica Aperio WebScope on average. A box plot of the quantitative evaluation result is provided in Fig. 4, showing that the proposed method works constantly well than Leica Aperio WebScope overall.

### X-direction test using a Tablet with wireless internet

**Figure 5:**
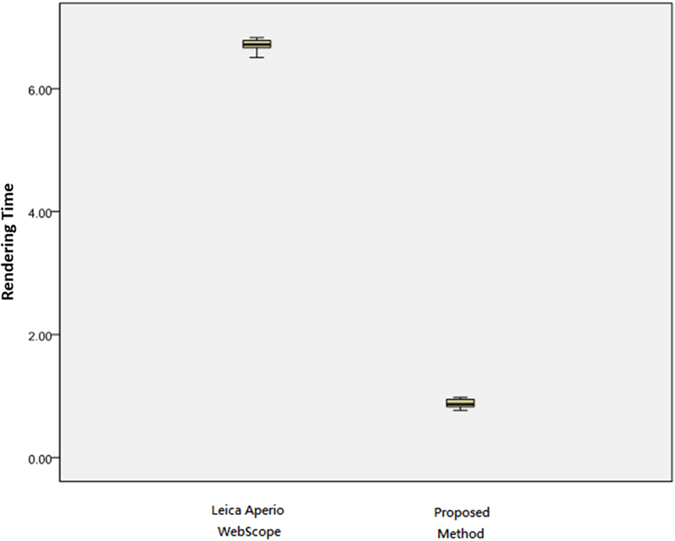
Rendering time by X-direction test using a Tablet with wireless internet. The rendering time of the proposed method performs faster than Leica Aperio WebScope about 7.603 times.

Table 7 presents the rendering time by X-direction test using a Tablet with wireless internet, and a statistical analysis using the Tukey’s HSD and the LSD tests is given in Table 8. The experimental results show that the proposed method achieves 0.88 seconds averaged rendering time and significantly outperforms the benchmark method on Tukey’s HSD and LSD tests (*P* ≤ 0.001). On average, it costs Leica Aperio WebScope 6.72 seconds, showing that the existing technique performs poorly and suffers from serious time delay. Overall, the proposed method performs 7.6 times faster than Leica Aperio WebScope on average. A box plot of the quantitative evaluation result is provided in Fig. 5, showing that the proposed method works constantly well than Leica Aperio WebScope overall.

Based on the experimental results, Table 9 further compares the ratios of the averaged rendering time by the proposed method to the benchmark techniques. In comparison to Microsoft HDview, the presented method is 94.13 times faster in the Z-direction test and 90.01 times faster in the X-direction test using a standard PC. On the other hand, in comparison to Leica Aperio WebScope, using a standard PC as the client site platform, the presented work is 20.35 times faster in the Z-direction test and 14.67 times faster in the X-direction test. Using a tablet with wireless internet, the presented work is 9.933 times faster in the Z-direction test and 7.603 times faster in the X-direction test than Leica Aperio WebScope.

## Discussion

As digital imaging technology advances, gigapixel or terapixel super resolution microscopic images become more and more common. However, due to the enormous size of these data, data analysis across sites or time is difficult because real time interactive accessing super resolution microscopic images over internet is challenging. Existing state of the art techniques such as Microsoft HDview and Leica Aperio WebScope suffer from significant time delay. We have developed a virtual microscopy method enabling real time interactive analysis of large-scale microscopic images through wireless or wired internet on standard desktops, laptops and mobile devices. VirtualMicroscopy enables analysis of super high resolution microscopic image across sites and time and allows multi-person analysis at the same time, which greatly speed up data analysis process and reduces miscommunication among scientists and doctors. A web site has been created for illustration purposes. (http://www-o.ntust.edu.tw/cweiwang/VirtualMicroscopy).

## Methods

**Figure 6:**
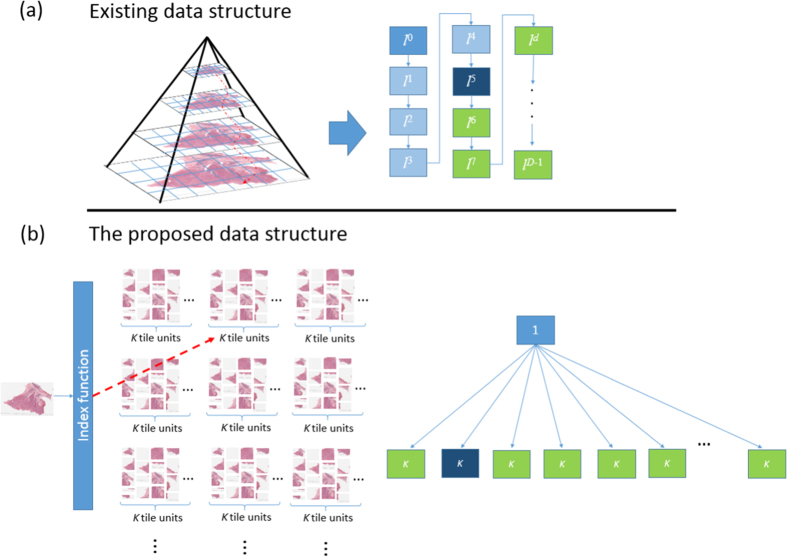
The difference between the existing pyramid data structure and the proposed tile units structure. The size of a set in the existing data structure requires *l*^*d*^, where *d* is the depth of the tier. Compared with existing pyramid data structure, the size of a set is limited at most *K* tiles (i.e. 2^8^).

Different from the commonly adopted pyramid data structure^11^,^12^,^13^,^14^,^15^,^16^ for large-scale images, a highly efficient quad-tree based data storage and accessing strategy is built in VirtualMicroscopy, enabling interactively accessing ultra-high resolution gigapixel or terapixel microscopic images, accessing gigapixel or terapixel data over internet in real time and interaction over internet with limited bandwidth in real time. In the common pyramid data structure^11^,^12^,^13^,^14^,^15^,^16^, a quad-tree model^17^,^18^ is used as the fundamental data structure. A quad-tree models a two-dimensional region by recursively dividing it into quadrants in the multi-resolution image tiers *d*. These quadrants are referred to as the leaves of the parent tile unit. Therefore, each parent tile unit has up to four leaf tile units. However, this model is a waste of time when seeking a unit locating in deep tiers. The search time of a tile unit in tier *d* requires *l*^*d*^, where *l* is set as four (quad-tree based), as seen in Fig. 6(a). Thus, we have designed a fast and efficient data structure for large data applications. The seek time of the proposed strategy is significantly reduced. In comparison to the existing data structure costing *l*^*d*^ times, the seeking time of the presented architecture is at most *K* as seen in Fig. 6(b), where *K* = 2^8^. For example, if the system is searching a unit in tier 11, *d* = 11, existing methods will take 4^11^ times. In comparison, the presented technique only takes at most 2^8^ times. The proposed data model for large data processing is presented as follows.

### Quickly data accessing strategy

The core idea behind the proposed framework is to access the regions of interest (ROI) directly and the neighboring tile units are saved in the neighboring physical address. Two key elements for quickly data accessing are the index table strategy and specific storage strategy.

#### Index table strategy

The main advantage of the index table over other data structures is speed. This advantage is more apparent when the number of tile units is large. Each tile unit is mapped into specific index code in the range 1 to *γ*, where *γ* denotes the total amount of tile units. The mapping is called an index function, which ideally should be simple to compute and should ensure that any two distinct tile units get different index codes. Then, the index codes are used to quickly locate the tile units without having to access each tier in the storage architecture.

The numbering of the tile name code is zero-based, therefore the smallest tier is represented by one tile unit that is at most *ST* × *ST* pixels with the tile code “0-0-0” and the index code “1”. Then, the codes for the index individual tiles can be generated as follows.

*Name code*: *z* − *y* − *x*, where *z* equals *d*, *x* is the number of the tile unit from left to right, and *y* is the number of the tile unit from top to bottom.


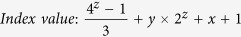


#### Specific storage strategy

The goal of the specific storage strategy is that the neighboring tile units are stored in the specific set. First, the size of each set is given as an integer *K*, the integer *K* should ideally be a power of two since it is easier to access in the computer architecture. Then, the second step is the specific storage ordering. Each tile unit is stored in hard drive according to the index code. The neighboring tile units of a tile unit will be stored in the same small set (*K* = 2^8^), therefore the seeking time for accessing neighboring tile units can be reduced to *K*.

Figure 6 shows the comparison of the proposed data structure and the existing data structure for ultra-high resolution images. For example, accessing a tile unit, which index code is 500, requires go through to tier five in the existing data structure; however, the proposed data structure only requires go through one tier for accessing the unit. According to the proposed specific storage strategy, the neighboring tile units will be stored in the same small set. On the contrary, a neighboring tile unit in the existing data structure may be sought in a larger set (i.e. with over than 2^10^ units). It means that it is wasting of time for seeking fundamental data units, which are stored in the deep tier. The reason is that the size of a set in the proposed data structure is limited by a small number *K* (*K* = 2^8^ is used in the experiments.). Therefore, the seeking time in a set can be reduced at most to *K*. In contrast to the existing data structure, the size of a set for deep tiers is larger and it requires significantly more seeking time. Table 10 presents the comparison of the proposed data structure and the existing pyramid data structure^11^, which is commonly used to deliver high-resolution images over the Web. In the proposed encoding and decoding algorithm, the proposed framework achieves great performance in the seeking time *O*(*k*). However, the seeking time for displaying a tile unit in the tier *d* requires *O*(*l*^*d*^), where the depth *d* ∈ [0, *D* − 1] in the existing data structure.

### Implementation

**Figure 7:**
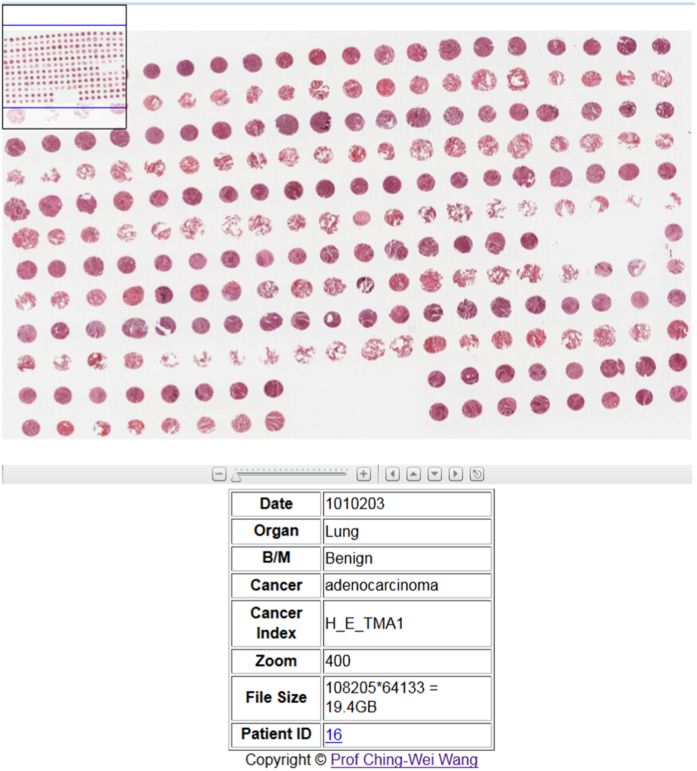
The Web interface of the proposed framework.

A demo website has been created for illustrating the presented Virtual Microscopy and Fig. 7 shows the web interface of the proposed framework. Demo website: http://www-o.ntust.edu.tw/~cweiwang/VirtualMicroscopy .

## Additional Information

**How to cite this article**: Wang, C.-W. *et al.* VirtualMicroscopy: ultra-fast interactive microscopy of gigapixel/terapixel images over internet. *Sci. Rep.* **5**, 14069; doi: 10.1038/srep14069 (2015).

## Supplementary Material

Supplementary video 1

Supplementary video 2

Supplementary video 3

Supplementary video 4

Supplementary Information - Video Legends

## Figures and Tables

**Figure 1:**
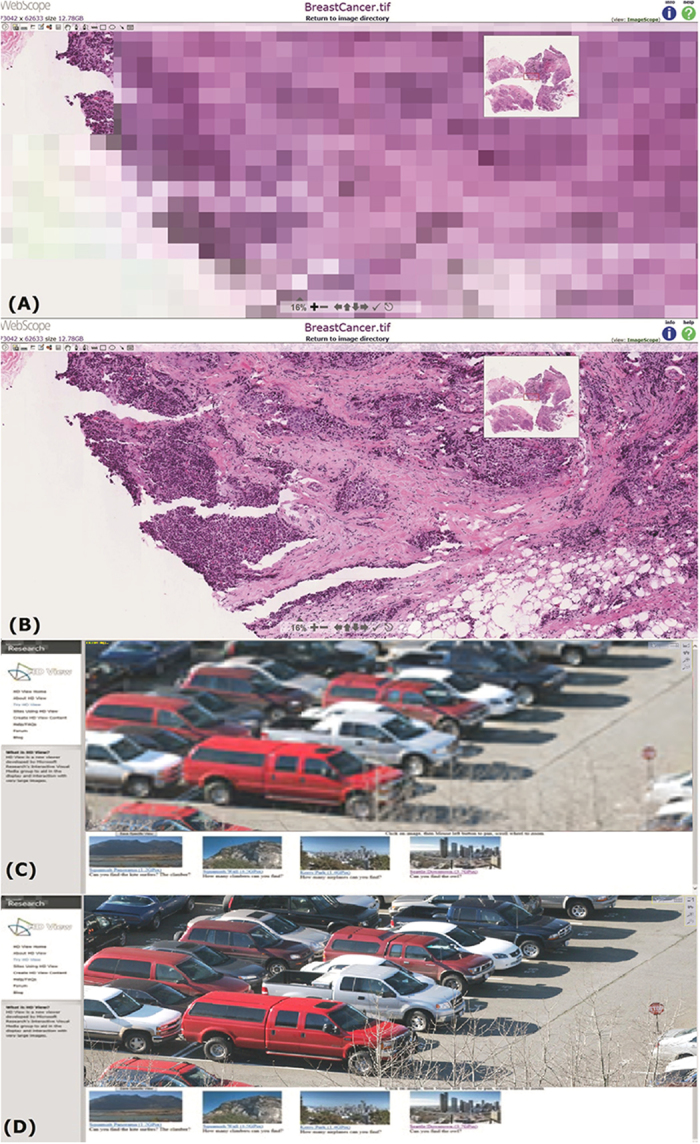
Existing techniques such as Microsoft HDview and Leica Aperio WebScope suffer from time delay and image blurriness. By interactively Z-direction test with 30 trials, (**A**,**B**) Leica Aperio WebScope^4^ costs 7.98 seconds on average for showing a 12.78 GB image, and (**C**,**D**) Microsoft HDview^3^ costs 36.9 seconds on average for showing a 3.7 GB image. Both suffer substantial delay. (Captured by co-authors, Chu-Mei Hun and Cheng-Ta Huang).

**Table 1:** Rendering time by Z-direction test using PC with wired internet.

Method	N	Mean (in sec.)	SD	Standard error
Microsoft HDview	30	36.897	2.44561	0.44651
Leica Aperio WebScope	30	7.978	1.06456	0.19436
Proposed Method	30	0.392	0.06769	0.01236

**Table 2:** Multiple comparison by Z-direction test using PC with wired internet: Tukey’s HSD and LSD.

(I) Method	(J) Existing Method	Mean difference (I-J)	Standard error	Sig.
Tukey HSD				
Proposed Method	Microsoft HDview	−36.50467*	0.28144	<0.001
	Leica Aperio WebScope	−7.58633*	0.28144	<0.001
LSD				
Proposed Method	Microsoft HDview	−36.50467*	0.28144	<0.001
	Leica Aperio WebScope	−7.58633*	0.28144	<0.001

**Table 3:** Rendering time by X-direction test using PC with wired internet.

Method	N	Mean (in sec.)	SD	Standard error
Microsoft HDview	30	32.67330	4.79683	0.87578
Leica Aperio WebScope	30	5.31700	0.42670	0.07791
Proposed Method	30	0.36270	0.06968	0.01272

**Table 4:** Multiple comparison by X-direction test using PC with wired internet: Tukey’s HSD and LSD.

(I) Method	(J) Existing Method	Mean difference (I-J)	Standard error	Sig.
Tukey HSD				
Proposed Method	Microsoft HDview	−32.31067*	0.50778	<0.001
	Leica Aperio WebScope	−4.95433*	0.50778	<0.001
LSD				
Proposed Method	Microsoft HDview	−32.31067*	0.50778	<0.001
	Leica Aperio WebScope	−4.95433*	0.50778	<0.001

**Table 5:** Rendering time by Z-direction test using a Tablet with wireless internet.

Method	N	Mean (in sec.)	SD	Standard error
Leica Aperio WebScope	30	13.84970	1.43483	0.26196
Proposed Method	30	1.39430	0.17520	0.03199

**Table 6:** Comparison by Z-direction test using a Tablet with wireless internet: Tukey’s HSD and LSD.

(I) Method	(J) Existing Method	Mean difference (I-J)	Standard error	Sig.
Tukey HSD				
Proposed Method	Leica Aperio WebScope	−12.45533*	0.16952	<0.001
LSD				
Proposed Method	Leica Aperio WebScope	−12.45533*	0.16952	<0.001

**Table 7:** Rendering time by X-direction test using a Tablet with wireless internet.

Method	N	Mean (in sec.)	SD	Standard error
Leica Aperio WebScope	30	6.71570	0.73050	0.01334
Proposed Method	30	0.88330	0.06671	0.01218

**Table 8:** Comparison by X-direction test using a Tablet with wireless internet: Tukey’s HSD and LSD.

(I) Method	(J) Existing Method	Mean difference (I-J)	Standard error	Sig.
Tukey HSD				
Proposed Method	Leica Aperio WebScope	−5.83233*	0.01882	<0.001
LSD				
Proposed Method	Leica Aperio WebScope	−4.67867*	0.16952	<0.001

**Table 9:** Ratios of the averaged rendering time by the proposed method to the benchmark techniques.

	Proposed Method: Microsoft HDview	Proposed Method: Leica Aperio WebScope
PC		
Z-direction Test	1:94.13	1:20.35
X-direction Test	1:90.01	1:14.67
Tablet		
Z-direction Test	x	1:9.933
X-direction Test	x	1:7.603

x: Microsoft HDview can not function on the Tablet with Android operating system.

**Table 10:** The comparison of the existing data structure and the proposed data structure.

	The Existing Data Structure	The Proposed Data Structure
The total amount of tiles in a set *d*	*l*^*d*^	*k*
Number of Sets	*log*_*l*_(1 + 3*γ*/*l*),	*γ*/*k*
Depth of Data Structure	*log*_*l*_(1 + 3*γ*/*l*)	2
Seeking time	*O*(*l*^*d*^)	*O*(*k*)

*l* is set as four, *d* denote the depth, where *d* ∈ [0, *D* − 1], *γ* denotes the total amount of the tiles and *k* denotes the size of each set.
