# Supplementary material for: VirtualMicroscopy: ultra-fast interactive microscopy of gigapixel/terapixel images over internet
Source: Sci Rep. 2015 Sep 11;5:14069. doi: 10.1038/srep14069 (PMC4566079; doi:10.1038/srep14069)
Supplement: Supplementary Information - Video Legends [file srep14069-s5.pdf]

Supplementary Information

VirtualMicroscopy: ultra-fast interactive microscopy of gigapixel / terapixel images over internet

Ching-Wei Wang[1,2,*], Cheng-Ta Huang[1] & Chu-Mei Hung[1]

[1]*Graduate Institute of Biomedical Engineering, National Taiwan University of Science and Technology, Taiwan*

[2]*Department of Biomedical Engineering, National Defense Medical Center, Taiwan.*

Video Captions

Video 1: Leica Aperio WebScope Interactive visualization of a super resolution image in the Leica Aperio WebScope Demo Website with image size 12.78 GB. The system suffers from significant time delay and image blurriness.

Video 2: Microsoft HDview Interactive visualization of a super resolution image in Microsoft HDview Demo Website with image size 3.7 GB. The system suffers from significant time delay and image blurriness.

Video 3: Demo video using PC with wired internet Interactive visualization of a super resolution microscopic image with image size 12.8 GB and image dimension 84570 × 54248 using the proposed method on a PC with wired internet.

Video 4: Demo video using a Tablet with wireless internet Interactive visualization of a super resolution microscopic image with image size 12.8 GB and image dimension 84570 × 54248 using the proposed method on a Tablet with wireless internet.
